# Real-World Variability in the Prediction of Intracranial Aneurysm Wall Shear Stress: The 2015 International Aneurysm CFD Challenge

**DOI:** 10.1007/s13239-018-00374-2

**Published:** 2018-09-10

**Authors:** Kristian Valen-Sendstad, Aslak W. Bergersen, Yuji Shimogonya, Leonid Goubergrits, Jan Bruening, Jordi Pallares, Salvatore Cito, Senol Piskin, Kerem Pekkan, Arjan J. Geers, Ignacio Larrabide, Saikiran Rapaka, Viorel Mihalef, Wenyu Fu, Aike Qiao, Kartik Jain, Sabine Roller, Kent-Andre Mardal, Ramji Kamakoti, Thomas Spirka, Neil Ashton, Alistair Revell, Nicolas Aristokleous, J. Graeme Houston, Masanori Tsuji, Fujimaro Ishida, Prahlad G. Menon, Leonard D. Browne, Stephen Broderick, Masaaki Shojima, Satoshi Koizumi, Michael Barbour, Alberto Aliseda, Hernán G. Morales, Thierry Lefèvre, Simona Hodis, Yahia M. Al-Smadi, Justin S. Tran, Alison L. Marsden, Sreeja Vaippummadhom, G. Albert Einstein, Alistair G. Brown, Kristian Debus, Kuniyasu Niizuma, Sherif Rashad, Shin-ichiro Sugiyama, M. Owais Khan, Adam R. Updegrove, Shawn C. Shadden, Bart M. W. Cornelissen, Charles B. L. M. Majoie, Philipp Berg, Sylvia Saalfield, Kenichi Kono, David A. Steinman

**Affiliations:** 1 Simula Research Laboratory and Center for Cardiological Innovation, Lysaker, Norway; 2 University of Oslo, Oslo, Norway; 3 Nihon University, Tokyo, Japan; 4 Charité – Universitätsmedizin Berlin, Berlin, Germany; 5 Universitat Rovira i Virgili, Tarragona, Spain; 6 University of Texas at San Antonio, San Antonio, TX USA; 7 Koc University, Istanbul, Turkey; 8 Universitat Pompeu Fabra, Barcelona, Spain; 9 Universidad Nacional del Centro de la Provincia de Buenos Aires, Buenos Aires, Argentina; 10 Siemens Medical Solutions USA Inc., Malvern, PA USA; 11 Beijing Union University, Beijing, China; 12 Beijing University of Technology, Beijing, China; 13 University of Siegen, Siegen, Germany; 14 University of Zürich, Zurich, Switzerland; 15 Dassault Systemes, Paris, France; 16 Simpleware Software Solutions, Exeter, UK; 17 University of Oxford, Oxford, UK; 18 University of Manchester, Manchester, UK; 19 University of Limerick, Limerick, Ireland; 20 University of Dundee, Dundee, UK; 21 Mie Chuo Medical Center, Tsu, Japan; 22 University of Pittsburgh, Pittsburgh, PA USA; 23 University of Tokyo, Tokyo, Japan; 24 University of Washington, Seattle, USA; 25 Medisys - Philips Research Paris, Paris, France; 26 Texas A&M University - Kingsville, Kingsville, TX USA; 27 Jordan University of Science and Technology, Irbid, Jordan; 28 Stanford University, Stanford, CA USA; 29 EinNel Technlogies, Chennai, India; 30 Siemens PLM Software, Plano, TX USA; 31 Tohoku University, Sendai, Japan; 32 Kohnan Hospital, Sendai, Japan; 33 University of Toronto, Toronto, ON Canada; 34 University of California, Berkeley, Berkeley, CA USA; 35 Academic Medical Center, Amsterdam, The Netherlands; 36 University of Magdeburg, Magdeburg, Germany; 37 Wakayama Rosai Hospital, Wakayama, Japan

**Keywords:** Intracranial aneurysm, Patient-specific modelling, Wall shear stress, Rupture risk, Uncertainty quantification

## Abstract

**Purpose:**

Image-based computational fluid dynamics (CFD) is widely used to predict intracranial aneurysm wall shear stress (WSS), particularly with the goal of improving rupture risk assessment. Nevertheless, concern has been expressed over the variability of predicted WSS and inconsistent associations with rupture. Previous challenges, and studies from individual groups, have focused on individual aspects of the image-based CFD pipeline. The aim of this Challenge was to quantify the total variability of the whole pipeline.

**Methods:**

3D rotational angiography image volumes of five middle cerebral artery aneurysms were provided to participants, who were free to choose their segmentation methods, boundary conditions, and CFD solver and settings. Participants were asked to fill out a questionnaire about their solution strategies and experience with aneurysm CFD, and provide surface distributions of WSS magnitude, from which we objectively derived a variety of hemodynamic parameters.

**Results:**

A total of 28 datasets were submitted, from 26 teams with varying levels of self-assessed experience. Wide variability of segmentations, CFD model extents, and inflow rates resulted in interquartile ranges of sac average WSS up to 56%, which reduced to < 30% after normalizing by parent artery WSS. Sac-maximum WSS and low shear area were more variable, while rank-ordering of cases by low or high shear showed only modest consensus among teams. Experience was not a significant predictor of variability.

**Conclusions:**

Wide variability exists in the prediction of intracranial aneurysm WSS. While segmentation and CFD solver techniques may be difficult to standardize across groups, our findings suggest that some of the variability in image-based CFD could be reduced by establishing guidelines for model extents, inflow rates, and blood properties, and by encouraging the reporting of normalized hemodynamic parameters.

## Introduction

Since the first individual case studies were published more than 15 years ago,^18^, ^22^, ^42^ medical image-based computational fluid dynamics (CFD) of intracranial aneurysms has become a widely-used tool for elucidating the role of hemodynamic forces in aneurysm development and rupture.^39^ Large retrospective studies (~ 200 cases) have shown associations between both low 47 and high 9 wall shear stress (WSS) and aneurysm rupture status, a seeming contradiction that may simply reflect a Janus-faced nature of aneurysm wall remodelling.^31^ On the other hand, it may also reflect the variability in the assumptions and compromises of aneurysm CFD studies, as well as inconsistent definitions of these (e.g., absolute vs. normalized) and other hemodynamic parameters associated with rupture.^5^, ^31^, ^36^

Image-based CFD is subject to numerous sources of uncertainty along its pipeline: the clinical modality used to image the aneurysm^4^, ^16^, ^17^; digital segmentation of the lumen, often requiring subjective decisions about thresholds, filtering, smoothing, *etc*.^15^, ^34^, ^38^; truncation of the domain and attendant assumptions about velocity boundary conditions^7^, ^19^, ^30^; the need to assume flow rates,^21^, ^25^, ^32^ since patient-specific measurements are rarely available; the pragmatic assumption of rigid walls 2, 12, 46 and simple blood rheologies^6^, ^27^, ^48^ when, similarly, patient-specific properties are difficult or impossible to obtain; and the choice of mesh and time-step resolutions, as well as other CFD solver settings.^13^, ^44^, ^45^ Common to the above-cited studies is that they were performed by individual research teams and focused on a single source of variability, all other factors being equal.

Triggered by a controversy in the clinical literature regarding a CFD-driven hypothesis about aneurysm treatment failures,^40^ a first International Aneurysm CFD Challenge was launched in 2012,^41^ focusing on a single giant internal carotid artery (ICA) side-wall aneurysm case. Participants were provided with the segmented lumen geometry, pulsatile flow rates, and blood properties, leaving the CFD solver and settings the only potential source of variability. Peak-systolic pressure drops were found to be predicted to within 8%, but peak-systolic velocity jetting into the sac turned out to be highly variable among the 27 CFD solutions submitted, including several that predicted flow instabilities where the rest did not. Closer, but not perfect, agreement was found for cycle-averaged velocity patterns.

A second Challenge was launched in 2013, to test whether, given two middle cerebral artery (MCA) bifurcation aneurysm cases, participants could identify the ruptured aneurysm, and also the site of rupture. In the first phase,^20^ 26 participating teams were provided with the segmented lumen geometry, requiring them to choose flow boundary conditions and blood properties. Despite a wide range of mesh densities, velocity boundary conditions and flow rates employed, all but five of the teams correctly identified the ruptured case, typically (but not exclusively) with low WSS as a determining factor; however, only one team correctly identified the rupture site. The organizers noted that the submitted WSS distributions had widely different magnitudes, so chose to display them normalized by their respective maximum WSS. Qualitative agreement was seen among most cases, but no quantification was provided. The organizers also noted, “[a]lthough some groups were highly experienced in other fields of engineering, the survey of the abstracts revealed that unrealistic inflow rates or velocities were applied. For instance, one group defined an inflow velocity of 10 m/s”.

In the second phase of the 2013 Challenge,^3^ participants were provided with flow rates and blood properties in order to narrow the source of variability to the CFD solution strategy alone. Centerline pressures and velocities showed generally good agreement, albeit with a handful of outliers, similar to what was seen in the first CFD Challenge.^41^ Velocity magnitudes on selected planes through the two models were also compared, showing that most groups captured the same flow patterns, and agreed to within about 20%.

In 2015, we (K.V.-S., K.K., and D.A.S.) decided to launch a third Challenge that would not only include more cases (five), but provide *no* information to participants beyond the source medical image volumes. The goal was twofold: (i) to test the ability to identify the ruptured cases, where the chances of guessing correctly was low, rather than 50% as in the previous Challenge; and (ii) to understand, for the first time, the total or “real-world” variability of aneurysm CFD. The results of the rupture prediction will be reported separately. The aim of the present study was to quantify the variability of image-based CFD predictions of aneurysm WSS when teams are left to choose their own segmentation methods, boundary conditions, blood properties, and CFD solution strategies.

## Methods

### Challenge Study Design

**Figure 1:**
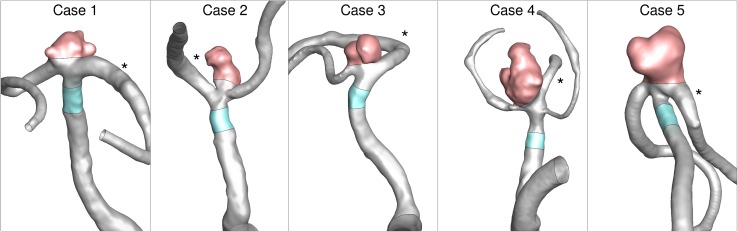
Representative segmentations of the five MCA aneurysm cases, showing the sac (pink) and parent artery (cyan) segments over which WSS was objectively averaged as described in the Methods. The * in each panel identifies dominant outflow branch, used to define the outflow division for all teams.

As shown in Fig. 1, five MCA bifurcation aneurysms were selected by one of the authors (K.K.) for having good 3D rotational angiography (3DRA) image quality, irregular shape, and similar size (~ 8 mm). The cases included a mix of ruptured and unruptured aneurysms; however, participants were blinded to rupture status. Challenge organizers confirmed that the five cases could be segmented and that CFD simulations could be carried out on the segmented models (those datasets were not included in the present study).

Teams were provided only with the DICOM image volumes, which included the ICA and the proximal and distal MCAs. Participants were free to choose their own segmentation methods, CFD solution strategies, flow rate and/or pressure boundary conditions, and material properties, mimicking real-world conditions for aneurysm CFD collaborations between clinicians and engineers. Among other relevant information, teams were asked to fill out a questionnaire with details on their solution strategy, and their self-assessed experience based on the number of aneurysm cases they had segmented and simulated: high (> 100 cases); medium (11–100 cases); low (1–10 cases); or none (0 cases). The questionnaire and the instructions sent to the teams are included in an online data repository.^1^ Teams were also asked to provide velocity and WSS fields (time-averaged and peak systolic for pulsatile simulations) for all five aneurysm cases. (Additionally, teams were asked to provide predictions of rupture status, and the geometric/hemodynamic parameters on which they were based; those results will be reported separately.)

### Response to the Challenge

A total of 45 teams registered for the Challenge, of which 26 provided CFD datasets including WSS fields. Two of these teams provided CFD datasets from two different segmentations; as discussed later, there were non-negligible intra-team differences, and so we treated these as independent submissions, resulting in 28 CFD datasets. Datasets from three teams were incomplete: Team 20 did not provide WSS or velocity fields for Case 5; Team 21 did not provide any velocity fields; and Team 24 provided the velocity field only for Case 1. Most teams provided velocity data as vector fields; however three teams (10, 13, 17) provided velocity magnitudes only.

### Centralized Data Analysis

Despite being derived from the same DICOM image volumes, the lumen geometries provided by the participating teams were in different scales, coordinate systems, rotations, and even mirrored. These were therefore first scaled to consistent units (mm) and mirrored if necessary. Centerlines were computed automatically from the lumen surfaces using the Vascular Modelling ToolKit (VMTK; www.vmtk.org), albeit with manual correction for some non-manifold surfaces. These were then initially registered automatically *via* the origin of the bifurcation hosting the aneurysm.^33^ Owing to the wide variability of the segmentations, surfaces were further manually rotated and translated to best match each other. The original and registered lumen surfaces, and the registered velocity and WSS fields are provided in the online data repository.^1^

Besides simplifying the visualisation of the multiple datasets, an advantage of registering the fields is that we could delineate a consistent segment of the parent artery (MCA) and the aneurysm sac using the same clipping planes for all teams. From the velocity datasets, lumen areas and mean through-plane velocities were calculated and averaged from five transverse slices (one slice for Case 4) through the MCA segment (c.f., cyan regions in Fig. 1). For the three teams that did not provide velocity *vectors*, we used their provided velocity magnitudes instead, after confirming that there was high correlation and no appreciable bias between velocities calculated from vectors vs. magnitudes from the other teams (*R*^2^ = 0.998, slope = 1.02).

### Parent Artery and Sac Hemodynamic Parameters

From the above areas and mean velocities we derived the parent artery diameters (assuming circular cross-sections), flow rates (area × velocity), Reynolds numbers (velocity × diameter × blood density/dynamic viscosity) and Poiseuille wall shear stress (32 × dynamic viscosity × flow rate/diameter^3^). Slices were also placed at a consistent location for each of the outlet branches in order to compute the flow rates, from which outflow divisions were determined. Again, it was confirmed that outflow divisions derived from velocity magnitudes were consistent with those from vector velocities (*R*^2^ = 0.985, slope = 0.97).

After clipping and isolating the aneurysm sac from the steady or time-averaged pulsatile WSS fields (c.f., pink regions in Fig. 1), we computed a trio of the simplest and arguably most-commonly-reported hemodynamic parameters^5^: AWSS, the sac-averaged WSS magnitude, in Pa; MWSS, the sac-maximum WSS magnitude,^9^ in Pa; and LSA, here defined as the surface area of the aneurysm sac exposed to WSS < 0.4 Pa and divided by the total sac area.^23^ A number of groups have also proposed normalizing these parameters to the parent artery WSS. After computing the average WSS magnitude over the clipped MCA segment, the following normalized hemodynamic parameters were computed: AWSS* = AWSS normalized by parent artery WSS^47^; MWSS* = MWSS normalized by the parent artery WSS^47^; and LSA*, the surface area of the aneurysm sac exposed to WSS < 0.1 × parent artery WSS, divided by the total sac area.^47^

Team characteristics and derived parent artery and sac hemodynamic parameters are provided in spreadsheet form in the online data repository.^1^ Teams are identified by their assigned ID number; however, certain information (country of origin, segmentation and CFD details) has been omitted in order to preserve team anonymity.

### Statistical Analysis

Almost all of the derived hemodynamic parameters did not have normal distributions according to D’Agostino & Pearson omnibus tests, and so are reported as median and interquartile range (IQR, the first (*Q*_1_) to third (*Q*_3_) quartile), with percent variability reported as the quartile coefficient of dispersion [CoD = (*Q*_3_ − Q_1_)/(Q_3_ + Q_1_)]. While most input parameters (flow rates, *etc*.) were found to be normally distributed, we chose to report them also using medians, IQR and CoD to be consistent with the statistics of the output hemodynamic parameters.

These descriptive statistics were calculated for each case individually, but also based on teams’ averages across the five cases, referred to as the “case-average” statistics. Where there might be missing data for one or more cases from a given team for a particular parameter, that team’s case-average value was not included. Kruskal–Wallis with *post hoc* Dunn’s tests were performed to determine whether significant differences in medians could be detected across aneurysm cases or experience levels, in light of variability. All statistical analyses were performed using Prism 6.0 (Graphpad Software, La Jolla CA), and significance was assumed at *p* < 0.05.

## Results

### Team, Solver, and Segmentation Variability

Per Table 1, there was a representative distribution of experience among the teams: 5 self-identified as highly experienced (> 100 cases) for both segmentation and CFD of cerebral aneurysms; 8 teams reported low or no experience (10 or fewer cases) with aneurysm segmentation or CFD; and the remaining 13 teams were somewhere in between. There was a good international distribution of teams, including high-experience teams from three continents.

**Table 1:** Summary of team/simulation characteristics.

	Experience^a^			
	High	Medium	Low	All
Number of teams	5	13	8	26
Continent^b^				
Europe	1.5	6.5	3	11
North or South America	1.5	3.5	4	9
Asia	2	3	1	6
Segmentation software^c^				
Mimics	2	2	1	5
VMTK	1	4	0	5
ITK-Snap	1	1	2	4
3D Slicer	0	1	2	3
Simvascular	0	0	2	2
Other	2	5	2	9
CFD software				
Fluent	3	4	1	8
CFX	2	2	0	4
Star-CCM+	0	0	3	3
OpenFOAM	0	2	0	2
Simvascular	0	0	2	2
Other	0	5	2	7
Rheology model				
Newtonian	4	13	6	23
Non-Newtonian	1	0	2	3
Viscosity (cPoise)				
3.5	3	5	4	12
3.7	0	1	1	2
4.0	2	7	3	12
Density (g/cm^3^)				
1.05–106	4	11	7	22
Other (1.0–1.05)	1	2	1	4
Temporal scheme				
Steady	4	7	4	15
Pulsatile	1	6	4	11
Inlet location				
MCA	0	11	6	17
ICA	5	2	2	9
Inflow scaling^d^				
Same flow rate (*n* = 0)	2	3	1	6
Same Re (*n* = 1)	0	1	1	2
Same velocity (*n* = 2)	1	6	3	10
Same WSS (*n* = 3)	2	1	1	4
Other	0	2	2	4
Inflow BC				
Plug	2	7	4	13
Poiseuille	3	3	2	8
Womersley	0	2	2	4
Other	0	1	0	1
Outflow BC				
Zero pressure	4	10	4	18
Cube (Murray’s) law	1	1	2	4
Other	0	2	2	4

^a^High: > 100 cases; Medium: 11–100 cases; Low: 10 or fewer cases

^b^Fractional values reflect teams split across continents

^c^Total = 28 since two teams used different software used for their two segmentations

^d^Power law relating flow rate to diameter, i.e., *Q* ~ *D*^*n*^

For CFD, more than half of the teams used a commercial solver, the rest using open-source or in-house codes. Interestingly, however, all high-experience groups used commercial (Ansys) solvers. The mesh resolution, distribution of cells in the domain, and local refinement, as well as solver settings, varied widely among teams, to the extent that objective comparisons were not attempted for the present study. All teams assumed rigid walls with no slip boundary conditions. Almost all teams assumed a Newtonian rheology, with blood density typically between 1.05 and 1.06 g/cm^3^, and viscosity almost equally divided between 3.5 and 4.0 cPoise (N.B., a 13% difference).

**Figure 2:**
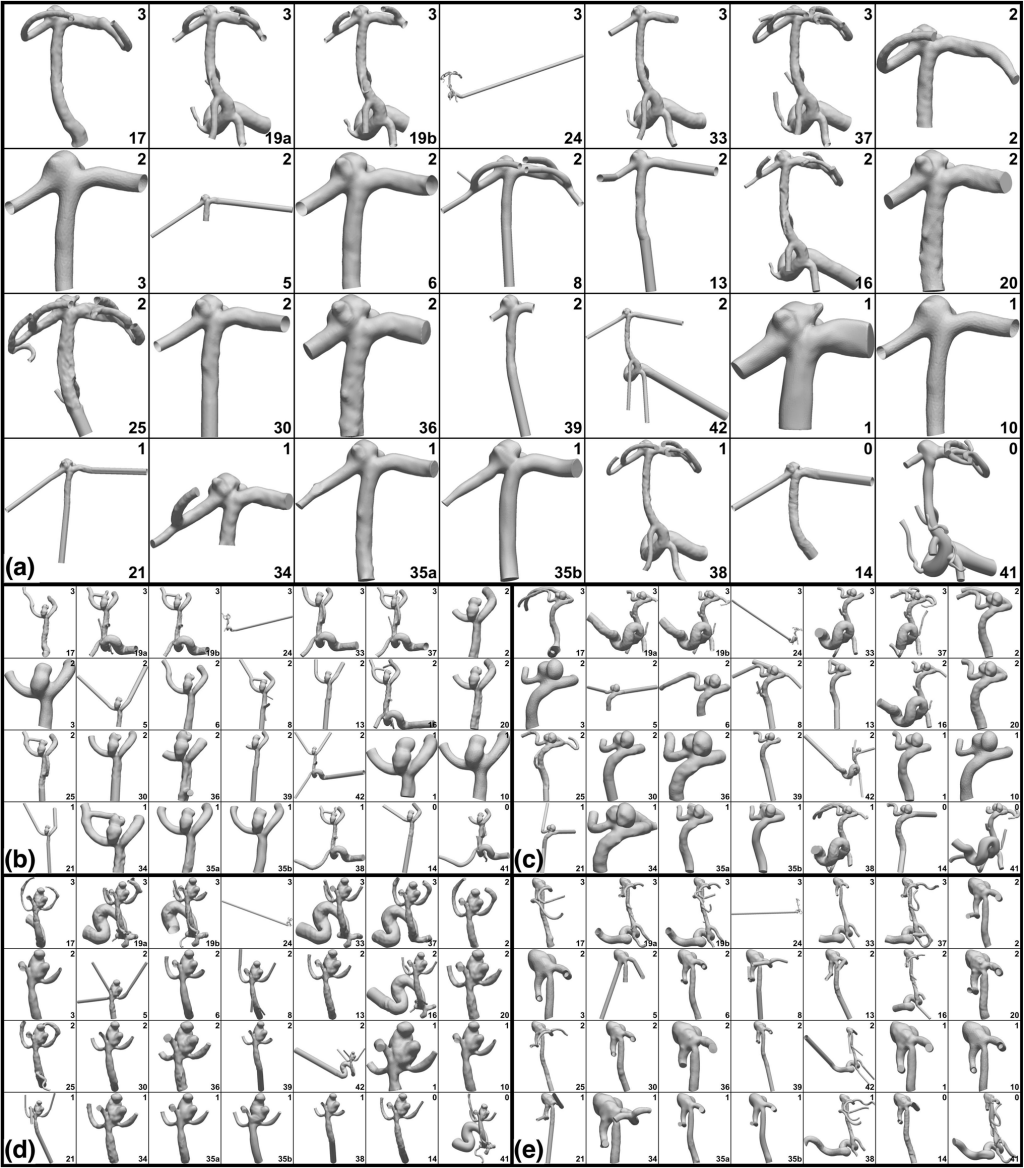
Variability of CFD model domains. (a) shows Case 1 at full size, while (b–e) show Cases 2–5 at reduced size in the interest of space. For each case, models are shown from top left to bottom right in descending order of team experience indicated in the top right corner of each panel: 3 = high; 2 = medium; 1/0 = low. Team number is shown at bottom right of each panel. For each case, models are all shown in the same view, but obviously not to the same scale.

A wide variety of software tools was used for segmentation, and these and other tools were also used for editing (smoothing, clipping, *etc*.) of the models. There was no obvious software preference based on experience level. Figure 2 shows the wide variability in segmentation and model extents, e.g., truncation of inlet at MCA vs. ICA, number and length of outflow and side branches, length of cylindrical flow extensions, *etc*. Notably, two-thirds of teams truncated their models at the MCA, and with varying lengths, while all high-experience teams included the ICA. The number of outlets (side or distal braches) also varied widely among teams.

**Figure 3:**
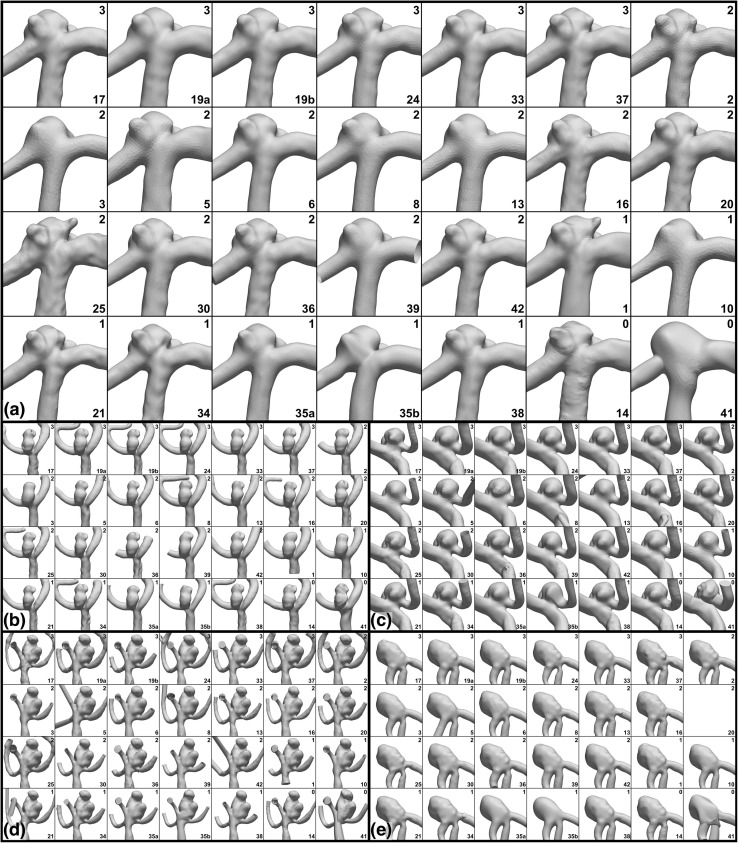
Variability of segmentations, focusing on the aneurysm and parent artery, with (a–e) showing Cases 1–5. Unlike Fig. 2, models are now zoomed in and, for each case, shown to the same scale in order to appreciate qualitative differences in sac and neck morphology, parent artery dimensions, and smoothness. As the surfaces are derived from the team-contributed WSS fields, mesh density may also be inferred from the faceting of the shaded surface. Experience levels and team numbers are shown in each panel, as explained in the caption of Fig. 2.

Taking a closer look at the aneurysms and parent arteries, Fig. 3 shows that, qualitatively and depending on the case, there could be wide variability in sac morphology and smoothness, neck size and location, and number and size of branches. For example, in Case 1 the number and size of the blebs was inconsistent, and there were clear differences in the diameters of the parent arteries (e.g., Team 3 vs. 5). For Case 2, the shape of the dome was highly variable, as were the neck location and width (e.g., Team 8 vs. 13). For Case 3 the width of the neck was also variable (e.g., Team 2 vs. 37), and although not visible in this view, so was the bottlenecking of the sac between two main lobes. For Case 4 the sac morphology and neck were more consistent, but the number and size of daughter branches was highly variable (e.g., Team 17 vs. 19a). For Case 5 the neck also appeared to be consistent among teams, but the degree of the stenosis proximal to the sac did not (e.g., Team 39 vs. 42).

**Figure 4:**
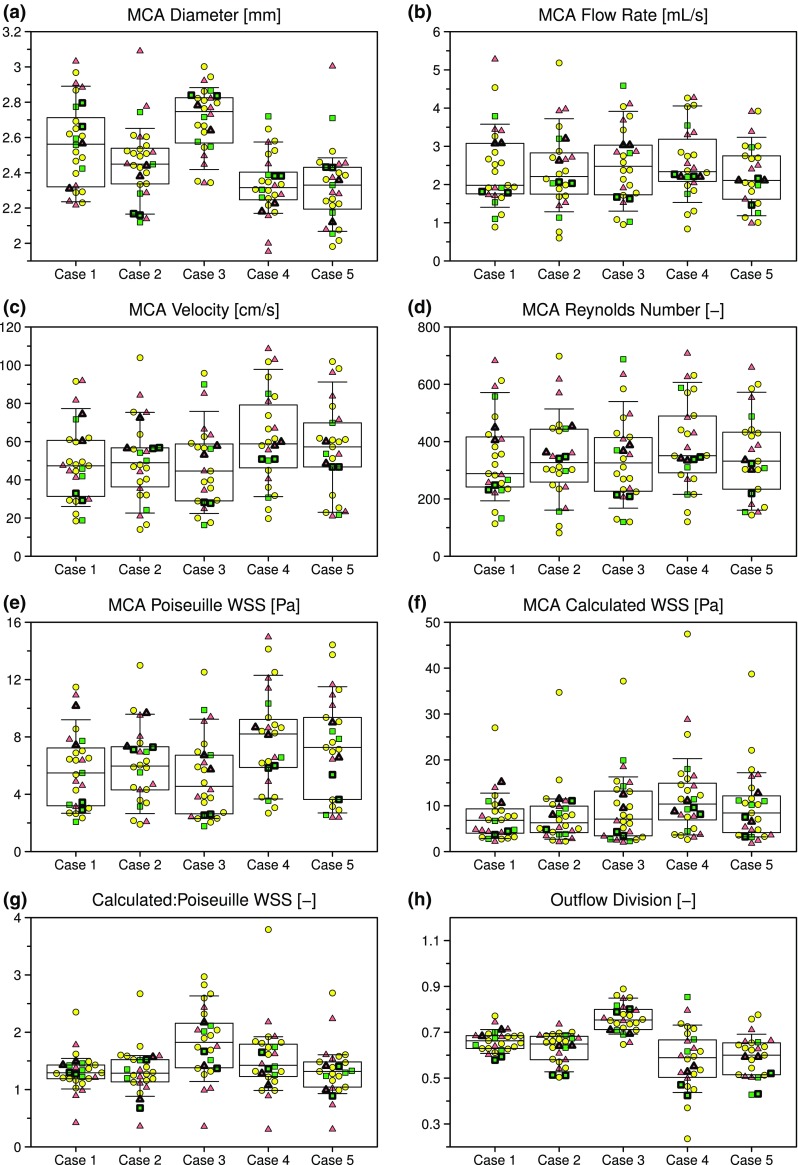
Variability of selected inflow/outflow parameters derived as described in the Methods. Green squares, yellow circles and red triangles identify data from teams with high, medium and low experience, respectively. Thicker symbols highlight the teams that contributed CFD datasets from two different segmentations. Superimposed horizontal lines, boxes, and whiskers identify median, IQR, and 90th percentile ranges for each case.

Table 2 and Fig. 4a show that, despite the variety of segmentation tools and techniques, and segmentation variability noted above, the MCA diameter, measured at a consistent location across teams, had a case-average CoD of only 3.4%, albeit up to 9% for Case 1 (N.B., which translates to CoD of 18% for cross-sectional area.). Significant differences in diameters for some of the cases could be detected (*p* < 0.0001), notably Cases 1–3 vs. Case 4 and 5. On average, variability was higher for low experience vs. medium or high experience teams; however, this was not true for individual aneurysm cases.

**Table 2:** Descriptive statistics for parent artery (MCA) inflow and outflow parameters, based on team case-average data.

Experience	N	Median	IQR	CoD (%)
Diameter (mm)				
All	27	2.45	2.40–2.56	3.4
High	6	2.50	2.39–2.56	3.5
Medium	12	2.47	2.40–2.58	3.4
Low	9	2.41	2.32–2.62	6.0
Flow rate (mL/s)				
All	25	2.40	1.82–2.91	23
High	5	1.99	1.63–2.81	27
Medium	12	2.30	1.88–2.95	22
Low	8	2.67	2.00–3.65	29
Velocity (cm/s)				
All	25	49.0	38.0–63.2	25
High	5	42.3	32.8–59.3	29
Medium	12	50.9	36.7–62.6	26
Low	8	59.0	40.1–76.8	31
Reynolds number (–)				
All	25	345	266–450	26
High	5	282	227–424	30
Medium	12	334	270–451	25
Low	8	376	288–535	30
Poiseuille WSS (Pa)				
All	25	6.19	4.48–8.31	30
High	5	4.91	3.91–7.16	29
Medium	12	6.48	4.11–7.61	30
Low	8	7.94	4.72–9.32	33
Calculated WSS (Pa)				
All	27	8.29	4.50–12.2	46
High	6	7.04	4.64–10.0	37
Medium	12	9.44	5.41–13.2	42
Low	9	6.51	4.05–12.9	52
WSS ratio^a^ (–)				
All	25	1.51	1.20–1.67	16
High	5	1.45	1.23–1.55	11
Medium	12	1.60	1.26–1.80	18
Low	8	1.37	1.03–1.64	23
Flow division (–)				
All	25	0.65	0.62–0.69	5
High	5	0.64	0.56–0.67	9
Medium	12	0.65	0.63–0.69	4
Low	8	0.65	0.62–0.70	6

^a^Ratio of Calculated:Poiseuille WSS

### Inflow and Outflow Variability

Since teams were challenged to carry out the CFD that *they* would require to predict rupture status, they were not obligated to assume pulsatile flows. In fact, just over half of the teams assumed steady flow conditions, including all but one of the high-experience teams. Of the 11 teams that used pulsatile simulations, waveforms were derived from a variety of sources (published vs. measured in-house vs. reduced-order model), vessels (common carotid artery vs. ICA vs. MCA), or cohorts (young adult vs. older adult vs. aneurysm patient).

The way in which steady or cycle-averaged flow rates were assigned by teams to the five aneurysm cases was also highly variable. Per Table 1, a plurality of teams (10/38%) assumed the same inlet velocity for all cases, which is tantamount to assuming that flow rate scales with inlet diameter squared (i.e., *Q* ~ *D*^2^). The next most common assumption (6/23%) was the same flow rate for all cases (*Q* ~ *D*^0^) followed by same WSS (*Q* ~ *D*^3^) and same Re (*Q* ~ *D*^1^). Even among the high-experience teams there was no consistency in the inflow scaling approach: two teams each assumed same WSS or flow rate, and one assumed same velocity. All but one of the 26 teams imposed their assigned inflow *via* Dirichlet (velocity profile) boundary conditions, the other team imposing pressure at both inlet and outlets. Inlet velocity profile shapes were almost equally distributed between plug and fully-developed (Poiseuille or Womersley), irrespective of experience level.

Per Table 2 and Figs. 4b–4g, the above variability in inflow strategies resulted in relatively wide variability in parent artery inflow characteristics. Flow rates varied by CoD = 23% on average, but up to CoD = 29% for Cases 3 and 5. As a result, there was no significant difference in median flow rates across the cases, nor was there a significant difference in medians due to experience level. This was also true for MCA velocities, which had case-average CoD = 25%, but up to 38% for Case 3; and for Reynolds number (Re), which had case-average CoD = 26%, and a maximum of 32% for Case 5.

The nominal (Poiseuille) inflow WSS, calculated from each team’s MCA diameter, flow rate, and blood viscosity/density, had a median value of 6.2 Pa (N.B., more than 4× the “normal” arterial WSS of 1.5 Pa^29^). The CFD-calculated inflow WSS, based on circumferentially averaging each CFD model over consistent parent artery segments (shown in Fig. 1), was higher at 8.3 Pa. Indeed, the median ratio of calculated:Poiseuille WSS was 1.5, and varied significantly (*p* = 0.007) from 1.3 (Cases 1, 2 and 5) to 1.8 (Case 3). Variability for calculated WSS, at CoD = 46%, was also higher than variability for Poiseuille WSS, at 30%. As such, while a significant difference in Poiseuille WSS between Cases 3 and 4 could be detected (*p* = 0.014), differences in calculated WSS could not. Variabilities for the ratio of Calculated:Poiseuille WSS ratio were lower (case-average CoD = 16%), suggesting that variability of calculated WSS among teams was driven more by differences in velocity *magnitudes* than velocity profile *shapes*. At the same time, among teams whose CFD models included the ICA siphon, the median ratio ranged from 1.3 to 1.7 among the cases, indicating that velocity profiles in the MCA cannot be assumed to be fully developed.

At the outlets, the majority of teams (18/69%), including all but one of the most experienced teams, assumed traction free conditions with zero pressure at all outlets. The second most popular approach (4/15%) was to divide outflows according to the cube of the diameter (i.e., Murray’s law), although it was not clear whether this was done explicitly with velocity profile (Dirichlet) or flux/pressure (Neumann) boundary conditions. The rest used either different scaling laws, reduced-order models, or did not specify. Despite the variability in outflow schemes, however, the division of outflow to the dominant branch was remarkably consistent (case-average CoD = 5%), with Case 4 having the highest variability (CoD = 16%) owing to the presence of three similarly-sized daughter branches (c.f., two branches for the other cases). As a result, there were significant differences (*p* < 0.0001) in median outflow divisions among some cases, notably Case 3.

### Wall Shear Stress Variability

**Figure 5:**
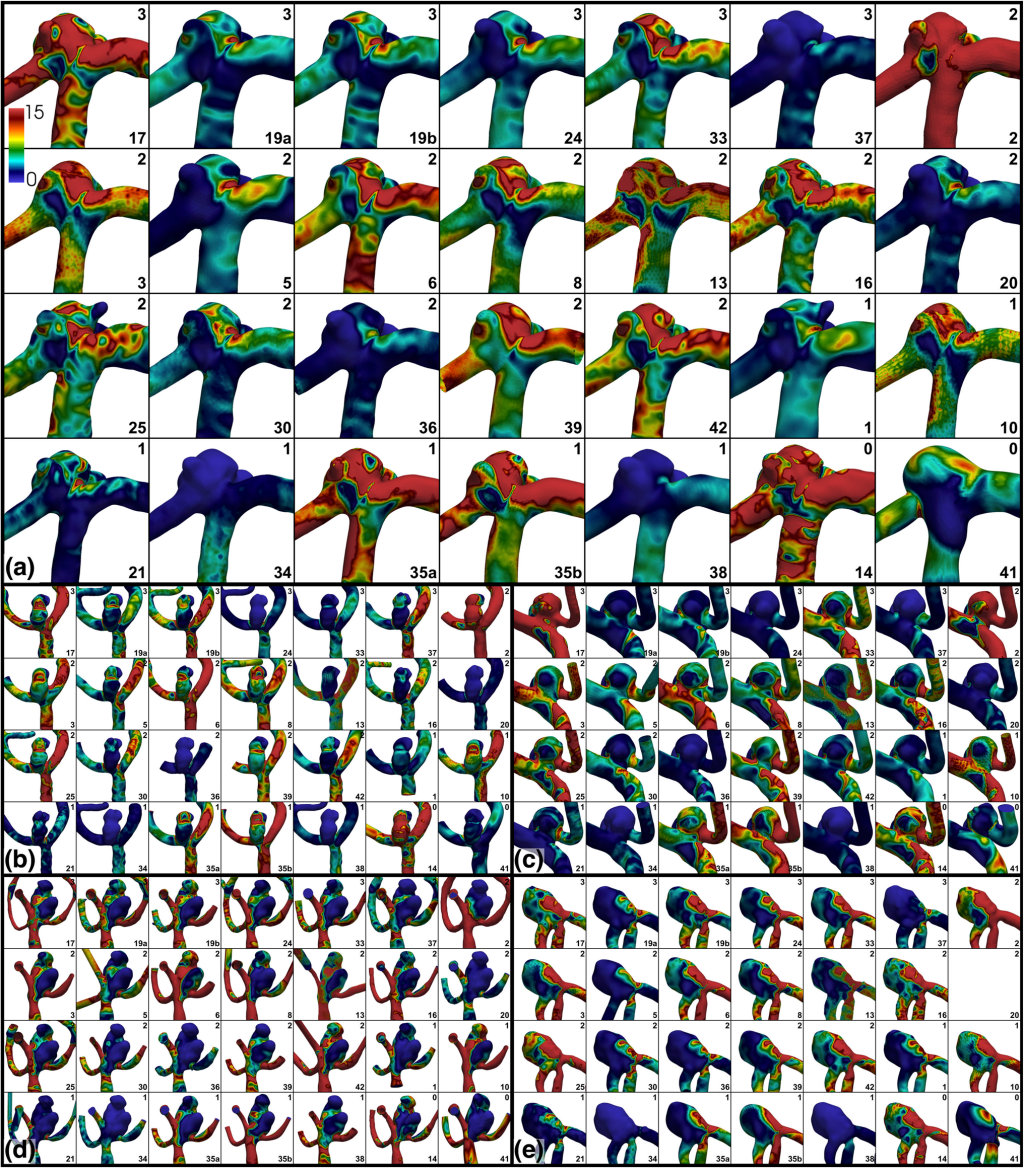
Variability of absolute WSS, with (a–e) showing Cases 1–5. WSS values are plotted from 0 to 15 Pa using the colour scale shown in the top left panels. Experience levels and team numbers are shown in each panel, as explained in the caption of Fig. 2.

**Figure 6:**
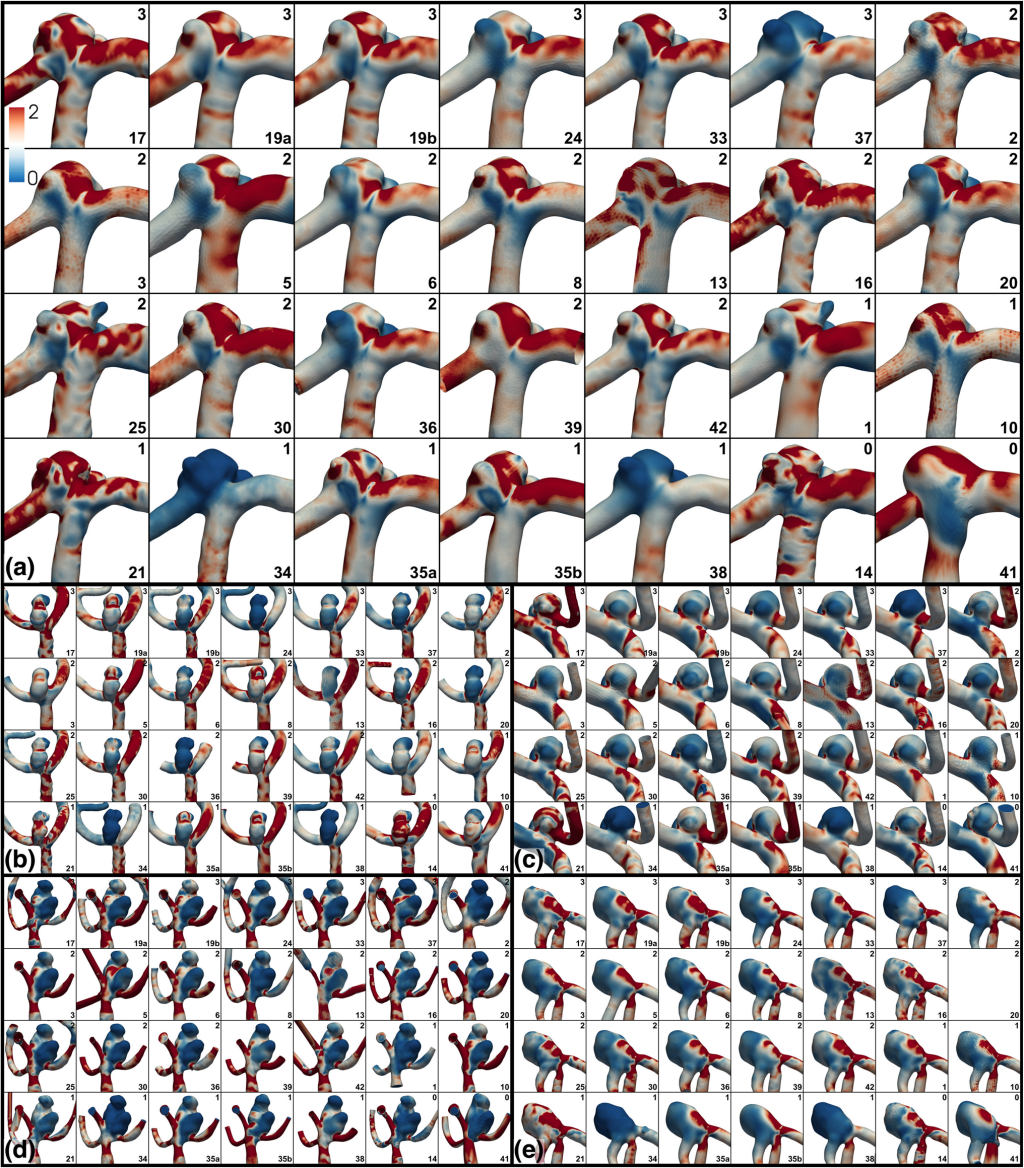
Variability of normalized WSS*, with (a–e) showing Cases 1–5. WSS* values are plotted from 0 to 2 using the colour scale shown in the top left panels, where WSS* = 1 corresponds to the nominal parent artery value. Experience levels and team numbers are shown in each panel, as explained in the caption of Fig. 2.

A qualitative overview of the variability of the computed WSS fields is presented in Fig. 5, demonstrating the wide differences in the magnitudes and spatial distribution of WSS, even among the most experienced teams. Indeed, the only consistency appears to be inconsistency among the teams. Figure 6 shows that a more consistent pattern of WSS emerges after normalizing by the parent artery (MCA) WSS, albeit still with sometimes appreciable differences in the location and extent of WSS extrema among teams, including among the most experienced teams.

**Figure 7:**
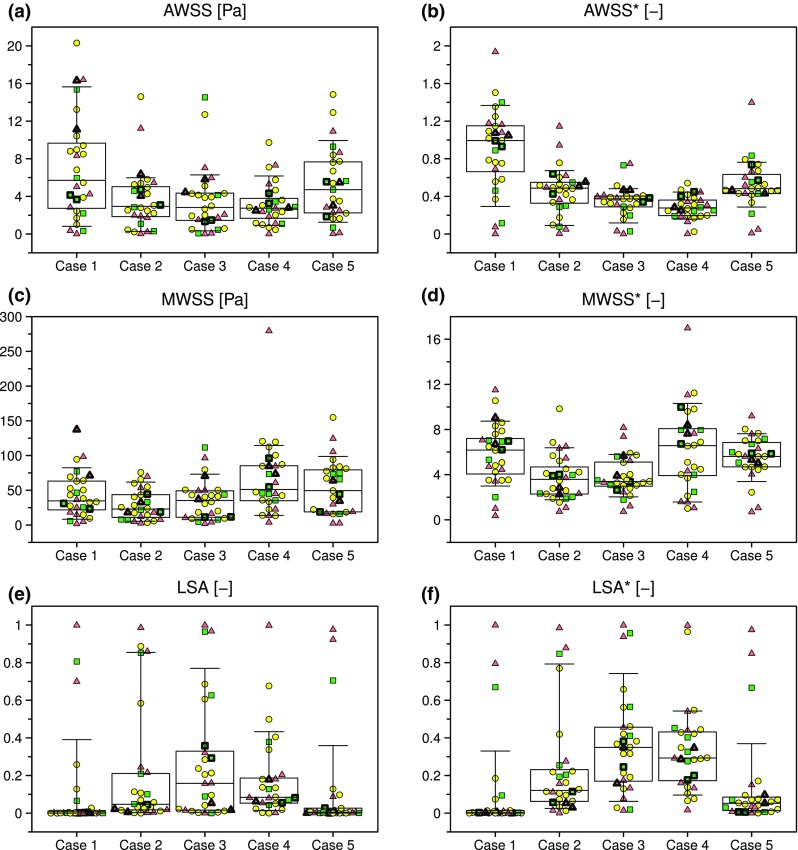
Variability of selected sac hemodynamic parameters derived as described in the Methods. See caption of Fig. 4 for explanation of symbols and box/whisker plots.

A more quantitative view of these results is presented in Table 3 and Fig. 7. Compared to the MCA inflow and outflow parameters shown in Table 2 and Fig. 4, there was, not surprisingly, more variability in hemodynamic parameters derived from the aneurysm sac. The most commonly reported parameter in the aneurysm CFD literature, sac-averaged WSS magnitude (here denoted AWSS), varied by CoD = 48% on average, but with CoD up to 60% for Case 1. There was no significant difference in case-averaged medians across aneurysm cases or experience levels. Case-average variability was reduced substantially after normalizing (i.e., AWSS*) to CoD = 18%, with a maximum CoD = 32% for Case 4 owing to its low median value. As a result, differences in medians across cases could be detected (*p* < 0.0001), notably between Cases 1 and 5 vs. 2–4.

**Table 3:** Descriptive statistics for aneurysm sac WSS parameters, based on team case-average data.

Experience	*N*	Median	IQR	CoD (%)
AWSS (Pa)				
All	27	4.57	2.24–6.31	48
High	6	3.26	1.83–5.40	49
Medium	12	5.63	2.91–6.44	38
Low	9	2.77	1.43–6.83	65
AWSS* (–)				
All	27	0.561	0.405–0.583	18
High	6	0.519	0.258–0.634	42
Medium	12	0.561	0.427–0.579	15
Low	9	0.559	0.271–0.649	41
MWSS (Pa)				
All	27	53.9	22.8–64.6	48
High	6	38.0	23.3–53.7	39
Medium	12	59.2	32.3–64.8	33
Low	9	34.5	16.2–69.4	62
MWSS* (–)				
All	27	5.41	3.83–5.94	22
High	6	5.21	4.09–5.53	15
Medium	12	5.58	3.99–6.37	23
Low	9	5.58	2.98–6.74	39
LSA (–)				
All	27	0.083	0.030–0.132	63
High	6	0.091	0.073–0.384	68
Medium	12	0.060	0.026–0.099	58
Low	9	0.052	0.022–0.431	90
LSA* (–)				
All	27	0.145	0.121–0.221	29
High	6	0.166	0.125–0.425	55
Medium	12	0.138	0.120–0.213	28
Low	9	0.153	0.097–0.475	66

Sac-maximum WSS (MWSS), being based on a point-wise rather than sac-averaged quantity, had ~ 10× higher IQR than AWSS; however, since the median MWSS was also ~ 10× higher, case-average CoD was identical to that of AWSS at 48%, albeit with three cases (2, 3 and 5) having individual CoD > 60% for MWSS. Case-average CoD for MWSS* was 22%, only slightly higher than 18% for AWSS*. Whereas for MWSS medians were only significantly different between Cases 2 and 4 (*p* = 0.003), for MWSS* Cases 1, 4, and 5 had significantly higher medians than Cases 2 and 3 (*p* < 0.0001).

Per Figs. 7e and 7f, LSA and LSA* both appeared to have similar variabilities to the other hemodynamic parameters, but as discussed later, had more apparent outliers. Case-average variabilities for LSA and LSA* were CoD = 63% and 30%, respectively, reflecting that, although both are dimensionless parameters, the threshold for low WSS is absolute for LSA, but relative to the parent artery for LSA*. CoD for individual cases were > 90% for both LSA (Cases 1, 3, and 5) and LSA* (Case 1), reflecting that the lowest quartile (Q_1_) value was close to 0. Nevertheless, despite these differences in case-average CoD between LSA and LSA*, and the high case-specific CoD, median LSA and LSA* were both significantly higher for Cases 2–4 vs. Cases 1 and 5 (*p* < 0.0001).

**Figure 8:**
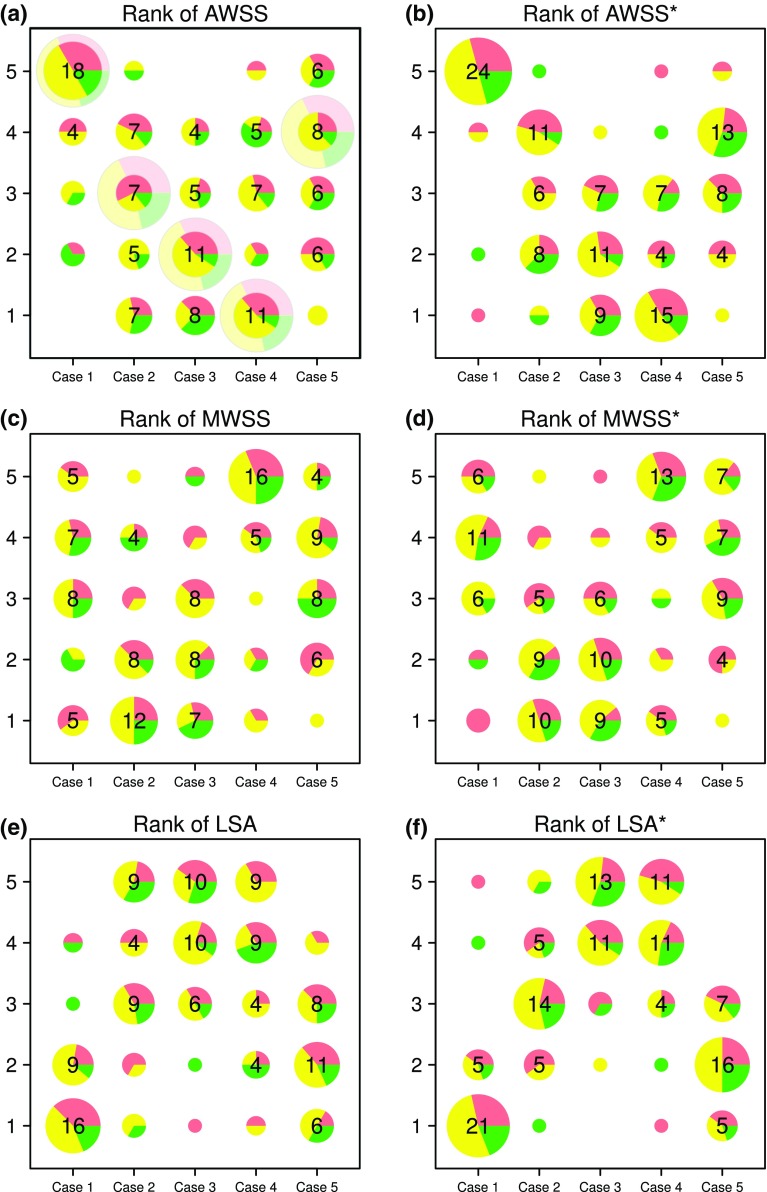
Variability of team rank-ordering of cases according the various hemodynamic parameters. In this bubble chart, the number of teams at each rank is proportional to the bubble area, while the proportion of high, medium and low experience teams at each rank is indicated by the green, yellow and red slices. The large, fainter bubbles in the top left panel indicate what one of these charts would look like for perfect agreement among all teams.

Finally, it could be imagined that, irrespective of differences in absolute values of a given hemodynamic parameter *between* teams, teams might be more consistent in terms of *rank*-*ordering* cases from low to high WSS. As shown in Fig. 8, rank-ordering did not eliminate variability, but it did seem to mitigate it. For dimensional hemodynamic parameters, consensus (i.e., more than half of teams) was reached only for Case 1 as having the highest-ranked AWSS and lowest-ranked LSA, and Case 4 having the highest-ranked MWSS. This could be seen as an improvement over absolute AWSS and MWSS as shown in Fig. 7, which because of the variability could not significantly discriminate a single case as having the highest value. Focusing on the normalized hemodynamic parameters, whereas AWSS* values shown in Fig. 7b could only significantly differentiate Cases 2–4 as low from Cases 1 and 5 as high, Fig. 8b shows that the majority of teams ranked Case 4 as having the lowest AWSS*, and nearly all teams ranked Case 1 as having the highest. Similarly, whereas MWSS* values in Fig. 7d could only identify significantly higher values for Cases 1, 4, and 5 vs. Cases 2 and 3, Fig. 8d showed that more teams ranked Case 4 as having the highest MWSS*. Finally, whereas LSA and LSA* could only significantly differentiate Cases 2–4 as high from Cases 1 and 5 as low in Figs. 7e, 7f, and 8e, 8f shows that the majority clearly identified Cases 3 and 4 as having the highest LSA and LSA* and Case 1 followed by Case 5 having the lowest.

## Discussion

### Summary of Key Findings

To the best of our knowledge, this Challenge presents the first report of the total (“real-world”) variability in aneurysm WSS as predicted by image-based aneurysm CFD, at least as practiced ca. 2015. It shows that there was appreciable variability in the prediction of aneurysm WSS, driven by the broad variety of strategies employed among participating teams for segmentation, boundary conditions, and CFD. Lumen geometries were highly variable in their morphology, extents and degrees of smoothing, yet while sac WSS magnitudes did vary substantially among teams (sometimes by orders of magnitude) there appeared to be more consensus regarding sac WSS patterns and relative ranking of cases after normalizing to the parent artery WSS.

Among the factors we could quantify objectively from the submitted data, input parameters like parent artery inflow rates and Reynolds numbers showed non-negligible case-average variabilities (23 and 26%, respectively), which resulted in variabilities of output hemodynamic parameters that could be higher (e.g., AWSS, 48%) or lower (e.g., AWSS*, 18%). The former is consistent with that fact that sac WSS should be proportional to flow rate, which is why normalizing to parent artery WSS, i.e., the latter AWSS*, typically reduces variability.

Since normalizing essentially renders the WSS *patterns* a function of the parent artery Reynolds number, it is interesting that high variability of Re resulted in lower overall variability of AWSS*. This echoes a point made at least as early as 2005,^8^ namely, that aneurysm flow *patterns* are relatively robust to variations in flow rate (i.e., Re). (However, see “Looking Beyond IQR and CoD”  section below for further discussion of this point.) This is encouraging in light of the fact that even good-faith estimations of inflow rates are probably in error relative to the actual—and usually unknown—patient-specific flow rates.^10^ With that said, we feel obliged to remind the reader that sac WSS dynamics, and especially high-frequency WSS fluctuations, may be more susceptible to variability in Re.^26^

Visually, there did not seem to be much difference in the variabilities of high vs. medium vs. low experience teams, which was reflected in the lack of significant differences in medians across experience levels. With the exception of the choice of solver (Ansys) and inlet location (ICA), high-experience teams did not show any more consensus about their image-based CFD pipelines than among other, less experienced teams.

### Intra-team Variability

Although the present study was not designed to systematically separate the influence of segmentation variability from boundary condition or solver variability, we note that two teams (19 and 35) each submitted two CFD datasets which differed only in terms of segmentation and/or smoothing, i.e., the inflow/outflow schemes and CFD solution strategies were the same within each team. For (high-experience) Team 19, automated vs. more intensive manual segmentations were performed, also with differences in the number and lengths of outflow branches. For (low experience) Team 35, two different segmentation software tools were used.

As reported in Table 4, segmentation generally had small influence on case-average MCA diameter, although for Team 35 differences could be as high as 11% for individual cases. Differences in case-average inflow characteristics were less than 10%; however, for individual cases, the imposed flow rate or Re could differ by as much as 38% (Team 19, Case 5). For Team 19, there was a 45% difference in case-average calculated MCA WSS between the two segmentations (driven by nearly 80% differences for Case 2 and 5), which is comparable to the *inter*-*team* CoD = 46% reported in Table 2. For Team 35, however, segmentation had a less dramatic, albeit still non-negligible (20%), effect on MCA WSS. Nevertheless, again for individual cases, MCA WSS could differ between segmentations by up to 65% (Case 5).

**Table 4:** Intra-team variability for input and output parameters, based on team case-average data.

Parameter	19a	19b	%diff^a^	35a	35b	%diff^a^
MCA diameter (mm)	2.52	2.49	1	2.38	2.42	2
MCA flow rate (mL/s)	1.84	1.99	8	2.72	2.61	4
MCA velocity (cm/s)	38.5	42.3	10	61.1	56.8	7
MCA Reynolds # (−)	270	294	9	385	362	6
MCA Poiseuille WSS (Pa)	4.44	4.91	10	8.25	7.63	8
MCA calculated WSS (Pa)	4.68	7.41	45	9.59	11.7	20
MCA WSS ratio (−)	1.11	1.51	30	1.16	1.57	30
MCA outflow division (−)	0.57	0.55	4	0.63	0.64	< 1
AWSS (Pa)	2.64	4.05	42	6.06	6.31	4
AWSS* (−)	0.597	0.577	4	0.559	0.550	2
MWSS (Pa)	25.5	45.5	56	60.2	64.6	7
MWSS* (−)	5.21	5.90	12	5.79	5.57	4
LSA (−)	0.090	0.091	1	0.045	0.024	60
LSA* (−)	0.103	0.136	28	0.122	0.153	22

^a^%diff = |*b* − *a*|/avg(*b* + *a*)

Absolute values of sac WSS differed appreciably between the two segmentations for Team 19 (42% for AWSS, 56% for MWSS, both driven largely by differences for Cases 2 and 5), but these were reduced to 4% and 12% by normalization, suggesting that much of this difference could be attributed to differences in parent artery (inflow) characteristics. For Team 35, sac WSS hardly differed between the two segmentations, except for a 60% difference in LSA, which could be attributed to its already-near-zero values. Taken together, these results indicate that even minor differences in segmentation may non-negligibly affect the commonly reported hemodynamic parameters, especially those based on absolute WSS, and thus *intra*-*team* variability may appreciably contribute to the *inter*-*team* variability.

### Reported Vs. Computed Quantities

As part of the Challenge, teams were asked to report their prescribed inflow rates and sac-averaged WSS for all five cases. Since some teams imposed inflow at the ICA, we were required to calculate parent artery (MCA) flow rates from their submitted velocity field data, as described in the Methods. For teams with MCA inlets, we also calculated their MCA flow rates from their CFD velocity fields, for quality control purposes.

**Figure 9:**
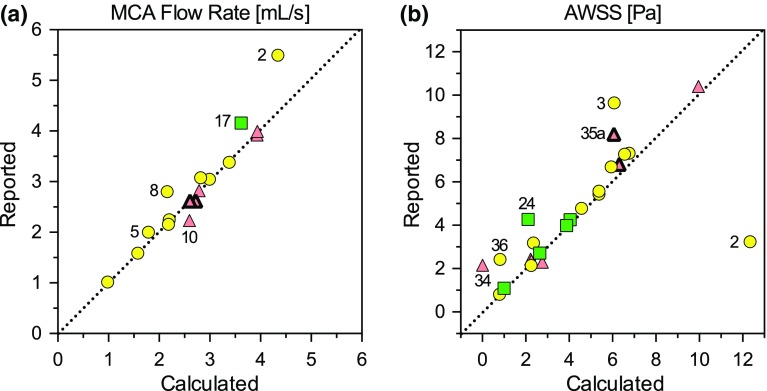
Comparison of calculated vs. reported quantities for (a) MCA flow rate and (b) sac-averaged WSS magnitude, i.e., AWSS. Data points are based on each team’s average across the five cases, and team numbers are shown for apparent outliers. See caption of Fig. 4 for explanation of symbols.

As Fig. 9a shows, there was generally excellent agreement between the reported and calculated MCA flow rates although, for 5 of the 16 teams that reported MCA flow rates, the calculated flow rates disagreed by more than 10%. For Team 8 this could be attributed to outflow from side branches included between the MCA inlet (where their reported flow rates were imposed) and the distal MCA (where our flow rates were calculated). Team 2 imposed plug velocity profiles on what turned out to be the coarsest tetrahedral meshes of any team, and without any boundary layer elements, so it is possible that the flow rates actually imposed may have been less than the nominal ones reported. Team 5 reported 2 mL/s for all five cases, but appear to have imposed 1 mL/s for Case 5. Regarding Teams 10 and 17, we note that they were among a handful of teams that did not submit vector velocity fields, requiring us to estimate flow rates from their provided velocity magnitudes rather than through-plane velocities we did for other teams; however, as noted in the Methods, this should not have introduced any significant bias.

Figure 9b shows that, for the 22 teams that reported their own AWSS values, there was generally good agreement with the AWSS that we calculated based on a consistent sac clipping plane, suggesting that the impact of sac delineation was generally negligible, at least for AWSS. Nevertheless, for a few teams (3, 24, 35a, 36) the reported AWSS averaged 1.5–3× higher than our calculated value. (Interestingly, Team 35’s other submission (35b) showed no such discrepancy). Conversely, Team 2 reported AWSS values that averaged about 4× *lower* than what we calculated from their WSS data. The largest discrepancy, however, was for Team 34, which reported AWSS averaging 2.2 Pa, but for which we calculated AWSS averaging 0.012 Pa from their WSS data, a nearly 200× difference. We initially suspected that this might be a discrepancy in the units of the WSS field provided, but their MCA WSS (calculated from the same WSS surface data) averaged 3.7 Pa, well within what other teams reported.

### Outlier and/or Inconsistent Data

According to published phase-contrast MRI measurements of nearly 100 adults, cycle-averaged blood flow rates in the MCA are 2.43 ± 0.52 mL/s,^50^ suggesting a 95th percentile range (i.e., roughly ± 2 SD) of 1.39–3.47 mL/s. Four teams (2, 14, 17, and 34) were up to 25% above this range, and one team (36) was 30% below. This may not, however, reflect a lack of experience—these teams had a mix of experience levels, from high to low—or knowledge of cerebrovascular flow rates. Three of the teams (2, 14, and 36) provided no specific rationale for their choice of flow rates; however, one team (34) did note that they chose to perform steady flow simulations corresponding to peak-systolic velocity conditions, which was not unreasonable in light of the focus of the Challenge on WSS variability in the context of predicting rupture status. On the other hand, for (high-experience) Team 17, CFD models were segmented proximal to the ICA terminus, but anterior cerebral artery (ACA) branches were not included. This team appeared to impose inflow rates consistent with those for the ICA, meaning that the one third of flow typically directed to the ACA^50^ was instead directed into the MCA.

These teams with outlier flow rates also tended to be outliers for hemodynamic parameters. Looking first at MCA WSS (Fig. 4f), Team 2 had values averaging 37 Pa, which was ~ 5× the median and ~ 2× higher than any other team. While this team did have the highest case-average MCA flow rates (4.34 mL/s), their predicted Poiseuille WSS of 12.8 Pa was not nearly as much of an outlier according to Fig. 4e. Instead, the high MCA WSS appears to have been due to this team’s use of plug velocity profile with a relatively short MCA inlet length, whereas most other teams with short MCA segments imposed fully-developed velocity profiles. On the other hand, Team 34, which similarly imposed plug velocity profiles onto CFD models with relatively short MCA inlet lengths, had comparable Poiseuille WSS (10.7 Pa), but, counter-intuitively, had *lower* MCA WSS values of only 3.7 Pa (in fact the only team for which this happened), further hinting at a possible inconsistency in the provided WSS surface data (more about this below).

Turning attention to Fig. 7, the highest AWSS was consistently provided by (medium experience) Team 2; however, their AWSS* values were comparable to those of other teams, which, as noted in the previous section, could be explained by Team 2’s high MCA WSS. At the other extreme, (low experience) Team 34 had AWSS averaging 0.012 Pa, ~ 400× lower than the median case-average AWSS. (This is not inconsistent with a recent meta-analysis, which reported ~ 100× differences in WSS levels across the aneurysm CFD literature.^5^) Consequently, this team’s LSA and LSA* values were also consistently outliers, close to 1.0. This would seem to suggest a possible inconsistency in the units of the provided WSS surface data, yet case-average MWSS for this team was 2.9 Pa, “only” ~ 20× lower than the median MWSS value.

This is not to say that only inexperienced teams contributed outlier results. Per Fig. 7a, one high-experience team (17) contributed some of the highest AWSS values for Cases 1 and 3, well in excess of any of the other high-experience team, likely due to their outlier high flow rates as discussed above. At the other end of the scale, Teams 37 (high experience) and 38 (medium experience) had AWSS values at least 5× lower than the median case-average AWSS, likely due to their flow rates (1.42 and 1.62 mL/s, respectively), which were at the low end of the spectrum. As a result, these teams were consistently among the outliers for LSA and LSA*. That rank-ordering of cases by the hemodynamic parameters (i.e., Fig. 8) improved consensus suggests that, even if a team over- or underestimated flow rates or WSS, as long as it was being done consistently, the *relative* ordering of cases by some WSS parameter could be more robust.

Finally, we do not mean to single out some of the above teams as the *only* outliers. Considering the 5 aneurysm cases and 14 (inflow, outflow, and sac) parameters investigated in the present study, *every* team had data points outside of the 10th–90th percentile range (i.e., “outliers”) for at least one of those 70 comparisons, and all teams were outside the IQR for at least 14 of those 70 comparisons. We do note, however, that low-experience teams contributed 43% of the “outlier” data points, compared to 40 and 17% from medium- and high-experience teams, respectively. This is out of proportion to the respective 32, 47 and 21% of all data points contributed by low-, medium- and high-experience teams, and would seem to suggest that, while we found no significant difference in the data across experience levels, low-experience teams were more likely to contribute outlier data.

### Looking Beyond IQR and CoD

In this study, we focused on IQR and CoD as standard descriptive statistics for datasets having non-parametric distributions. This however, makes it more difficult to compare against the standard deviations (SD) and coefficients of variation (i.e., CoV = SD/mean) typically reported in the literature (albeit often without testing for normality). To give some context, CoD was 23% for case-averaged MCA flow rates, which could be considered negligible or at least tolerable in light of an early report that ± 25% variations in flow rate had only a modest impact of aneurysm flow patterns.^8^ This, however, ignores that fact that IQR and CoD include, by definition, only half of the 28 datasets.

Expanding to the 10th and 90th percentiles (the “whiskers” in Figs. 4 and 7) brings in 22 of the 28 datasets. The resulting *inter*-*decile* range for MCA flow rates is 2.2×, greater, corresponding to a percent variability of 44%. Similarly, for case-averaged AWSS and AWSS*, the inter-decile ranges were 2.2× and 3.1× wider than their respective IQRs, corresponding to percent variabilities of 85 and 63%, vs. their respective CoDs of 48 and 18%. We therefore recommend some caution in relying solely on IQR and CoD as measures of variability, since they will tend to paint a more optimistic picture of the breadth of the variability. A good rule of thumb for our data would seem to be that 2 × IQR or 2 × CoD encompass the variability of most teams.

### Caveats

As noted in the Introduction, the aim of this Challenge was decidedly *not* to separate the impact of the various (and often interacting) input variabilities on output hemodynamic parameters. We attempted this only where we could objectively characterize input parameters like inflow rates or outflow divisions. Those findings seemed to suggest a prominent role for inflow variability on the variability of the chosen hemodynamic parameters, but we cannot say with authority to what extent segmentation or CFD solver/settings variability may have contributed. We also cannot say to what extent inlet location vs. choice of inflow power law may have impacted the variability in prescribed flow rates.^43^ Finally, in choosing a consistent location for the parent artery segment, from which derived the MCA velocity, Re, and normalizing WSS, we obscured a potential contribution to the real-world variability in those input parameters, and in the normalizing of absolute hemodynamic parameters.

Because of the underlying objective of understanding CFD variability in the context of rupture status/risk assessment, we did not require pulsatile simulations, and focused only on the most-common integrated or point-wise hemodynamic parameters, for which steady flow is anyway considered a good proxy for time-averaged pulsatile flow.^35^ Thus, our findings cannot be extrapolated to applications where the spatiotemporal fluctuations of WSS may be of interest, e.g., oscillatory shear index (OSI),^49^ spectral power index,^26^*etc*. In those cases, the impact of flow rate pulsatility (and CFD solver settings 28) cannot be overlooked, especially since, as noted in the “Results”, teams that did perform pulsatile CFD employed a wide variety of flow waveform shapes.

We also remind the reader that the reported variabilities are predicated on medians derived from the submitted teams; however, it is not at all clear that the majority should rule. First, while the 26 teams span a wide range of expertises and strategies, their distribution may not be representative of the aneurysm CFD community or published studies as a whole. For example, our Challenge did not attract participants from some of the most well-published aneurysm CFD groups. Second, what constitutes “truth” in image-based aneurysm CFD remains an open question.^24^ Even if we were to eliminate variability in segmentations, boundary conditions and CFD solutions, medical imaging can introduce its own distortions, and patient-specific input parameters like flow rates are usually not known, and are anyway subject to their own inherent physiological variations.

Finally, although this Challenge did involve a large amount of data, it was still based on “only” five aneurysms of bifurcation type from a particular cerebrovascular territory. Some caution must therefore be exercised before extrapolating these findings too broadly.

## Conclusions

Wide variability exists in the prediction of intracranial aneurysm WSS, irrespective of experience with image-based aneurysm CFD. This serves as an impediment to the integration of studies from different groups,^5^ a step that may be required in order to achieve statistically significant findings in light of the many factors, other than hemodynamic forces, that influence aneurysm growth and rupture.^37^

Segmentation appears to introduce variability in two ways: (i) morphology and smoothness of the aneurysm sac, neck and parent artery region; and (ii) inconsistent model extents, making the CFD models more sensitive to inflow and outflow boundary conditions. The impact of the former we can only speculate about, and we appreciate that consensus may be difficult to achieve regarding segmentation methods. (The Multiple Aneurysms Anatomy Challenge (MATCH), announced in early 2018, may help at least address the question of how segmentation variability affects output hemodynamic parameters, since the organizers intend to perform their own consistent CFD on segmentations of five aneurysms provided by the participating teams.) Regarding the latter, our study showed that fully-developed flow was not present in the MCA even when it was far downstream of the (ICA) inlet, suggesting that clipping of the parent artery to within a few diameters of the aneurysm should be strictly avoided. Instead, as previous studies have intimated,^7^, ^19^ segmentations should include as much of the proximal vasculature as possible in order to help minimize this unnecessary source of variability.

Inflow rates were demonstrably variable and appeared to drive at least some of the variability among the CFD solutions. While patient-specific flow rates are rarely known, and are anyway subject to normal physiological variability within a given patient, some unnecessary variability in aneurysm CFD may be introduced by the use of outlier flow rates. When patient-specific flow rates are not available, sanity checks on estimated inflow rates and Reynolds numbers can and should be performed against literature values and ranges. Outflow boundary conditions here appeared to have only a minor impact on the variability of outflow divisions, although it is hard to know whether and how these might impact flow and WSS patterns for individual aneurysms,^11^ or for cases where more extensive outflow tracts may be included.

Blood properties were also likely a relatively minor source of variability, although differences in input parameters could, in principle, be up to 13% just by virtue of the almost even split between teams using blood viscosities of 3.5 and 4.0 cPoise. While blood properties do vary from patient to patient, and also within patients, this information is not always easily available clinically, especially for retrospective studies. Instead, when patient-specific properties are not available, we suggest that this source of variability, whatever its influence on aneurysm CFD, could easily be removed by standardizing values. We recommend a dynamic viscosity of 3.7 cPoise, which falls neatly between the values that teams typically used, and, with a recommended standard density of 1.06 g/cm^3^, yields a nice round number of 3.5 cStokes for kinematic viscosity.

In this study we did not attempt to separate the influence of CFD solution strategy in light of the many other uncontrolled sources of variability. While studies have shown that CFD solver and mesh/timestep resolutions can have a non-negligible impact on the values of hemodynamic parameters based on point-wise (e.g., MWSS) or time-dependent WSS (e.g., OSI),^14^, ^28^ stratification of cases by time-averaged and/or normalized hemodynamic parameters (e.g., AWSS* or MWSS*) may be more robust to CFD discretization or solver settings, all other factors being equal.^44^ We may therefore speculate that CFD solution strategy was a relatively minor source of variability in the present study.

Finally, our findings show that, whatever the relative contribution of the above-noted individual sources of variability may be, hemodynamic parameters based on normalized rather than absolute WSS have lower variability as a whole. This would seem to suggest that such parameters should be standardized and adopted more widely, at least until we understand better the biological and clinical implications of absolute vs. relative WSS.

In closing, we note that we have only scratched the surface in terms of the analyses that could be done with the rich datasets collected by this Challenge, and so we encourage others to explore the interactions among solution strategies, geometry and hemodynamics using the raw data, surfaces, velocity fields and WSS fields provided in the online data repository.^1^
